# Interrater reliability for unilateral and bilateral tests to measure the popliteal angle in children and youth with cerebral palsy

**DOI:** 10.1186/s12891-021-04135-6

**Published:** 2021-03-13

**Authors:** Erika Cloodt, Joanna Krasny, Marek Jozwiak, Elisabet Rodby-Bousquet

**Affiliations:** 1 Department of Clinical Sciences Lund, Orthopaedics, Lund University, Lund, Sweden; 2 Department of Research and Development, Region Kronoberg, Växjö, Sweden; 3 Department of Pediatric Orthopedics and Traumatology, Poznan University of Medical Sciences, Poznan, Poland; 4 Centre for Clinical Research, Uppsala University-Region Västmanland, Västerås, Sweden

**Keywords:** Cerebral palsy, Hamstring muscles, Physical examination, Range of motion, Reproducibility of results

## Abstract

**Background:**

Short hamstring muscles can cause several problems for children with cerebral palsy. The results of the clinical measurement of hamstring length are often used in decision-making about treatment of children with cerebral palsy. There are different ways of performing this measurement. The aim of this study was to evaluate the interrater reliability of the unilateral and bilateral measurement of the popliteal angle in children and youth with cerebral palsy.

**Methods:**

Two methods for estimating hamstring length using unilateral and bilateral measurements of the popliteal angle were applied in children with cerebral palsy. Both tests were applied bilaterally by two independent examiners on the same day for each child. The intraclass correlation coefficient (ICC) was calculated to evaluate the interrater reliability of both measurements. Seventy young people with cerebral palsy (32 females, 38 males, mean age 10 years 8 months, range 5–22 years) at Gross Motor Function Classification System levels I (*n* = 17), II (*n* = 31), III (*n* = 12) and IV (*n* = 10) were included.

**Results:**

The interrater reliability was good for both measurements. The ICC values were 0.80 on the right and 0.86 on the left for the unilateral popliteal angle, and 0.82 on the right and 0.83 on the left for the bilateral popliteal angle.

**Conclusions:**

Both unilateral and bilateral measurement of the popliteal angle is a reliable method for estimating hamstring length in children and youth with cerebral palsy.

## Background

Reduced muscle length is a common problem among children with cerebral palsy because of the effects of this non-progressive brain disorder and secondary problems such as spasticity, immobility, pathological muscle growth and fewer satellite cells compared with children without cerebral palsy [1–3]. Short hamstring muscles can cause gait problems for ambulatory children and can lead to postural asymmetries in standing and lying for non-ambulant children. The hamstring muscles semitendinosus, semimembranosus and biceps femoris (long head) are two-joint muscles that cross both the hip and knee joint, and are active in hip extension and knee flexion movements. Short hamstrings can cause the “crouch gait” and can reduce knee extension during the stance phase of walking in children with cerebral palsy [4, 5].

Two of five gait patterns described for unilateral cerebral palsy and three of four described for bilateral cerebral palsy involve limited knee extension and potential short hamstring muscles. The gait pattern can be described as equinus, jump knee and crouch gait [6]. Shortened hamstrings are strongly associated with a high risk for knee contracture, and muscle length has a stronger impact on knee contracture development than the level of spasticity [7]. Shortened muscle length of the hamstrings is a common reason for surgery in children with cerebral palsy [8, 9]. Hamstring lengthening is performed to improve gait and/or standing [4, 10].

The average routine for estimating hamstring length in the clinic is the goniometric measurement of the passive range of motion of the knee joint with the patient in the supine position and the hip in flexion [11]. There are different ways of performing this test, two of which are unilateral and bilateral measurements of the popliteal angle. The bilateral popliteal angle test is also called the 90–90 straight-leg test in the literature [12]. The results of the clinical measurement of hamstring length are often used in decision-making about treatment and when evaluating the treatment of children with cerebral palsy [9, 13, 14]. Given the lack of other convenient methods for estimating hamstring length in daily clinical practice and the need for this information for decision-making, it is important to know the reliability of the measurements in current use.

The aim of this study was to evaluate the interrater reliability of unilateral and bilateral tests to measure the popliteal angle when used in children and youth with cerebral palsy.

## Methods

The popliteal angle was measured using the unilateral and bilateral tests in 70 children and youth with cerebral palsy. All children were recruited at a rehabilitation centre, between 10 June and 2 September 2019. The inclusion criterion was diagnosis of cerebral palsy. The exclusion criterion was unilateral cerebral palsy to ensure that no unaffected legs were included. All measurements were performed independently by two physiotherapists on the same day for each child. Both the left and right legs were measured, and the order of measurements was randomized for the two examiners. Before the start of the study, the examiners were instructed in performing the two tests using a goniometer and a standardized method. Both physiotherapists had recently graduated and had no previous experience measuring children with cerebral palsy.

**Fig. 1:**
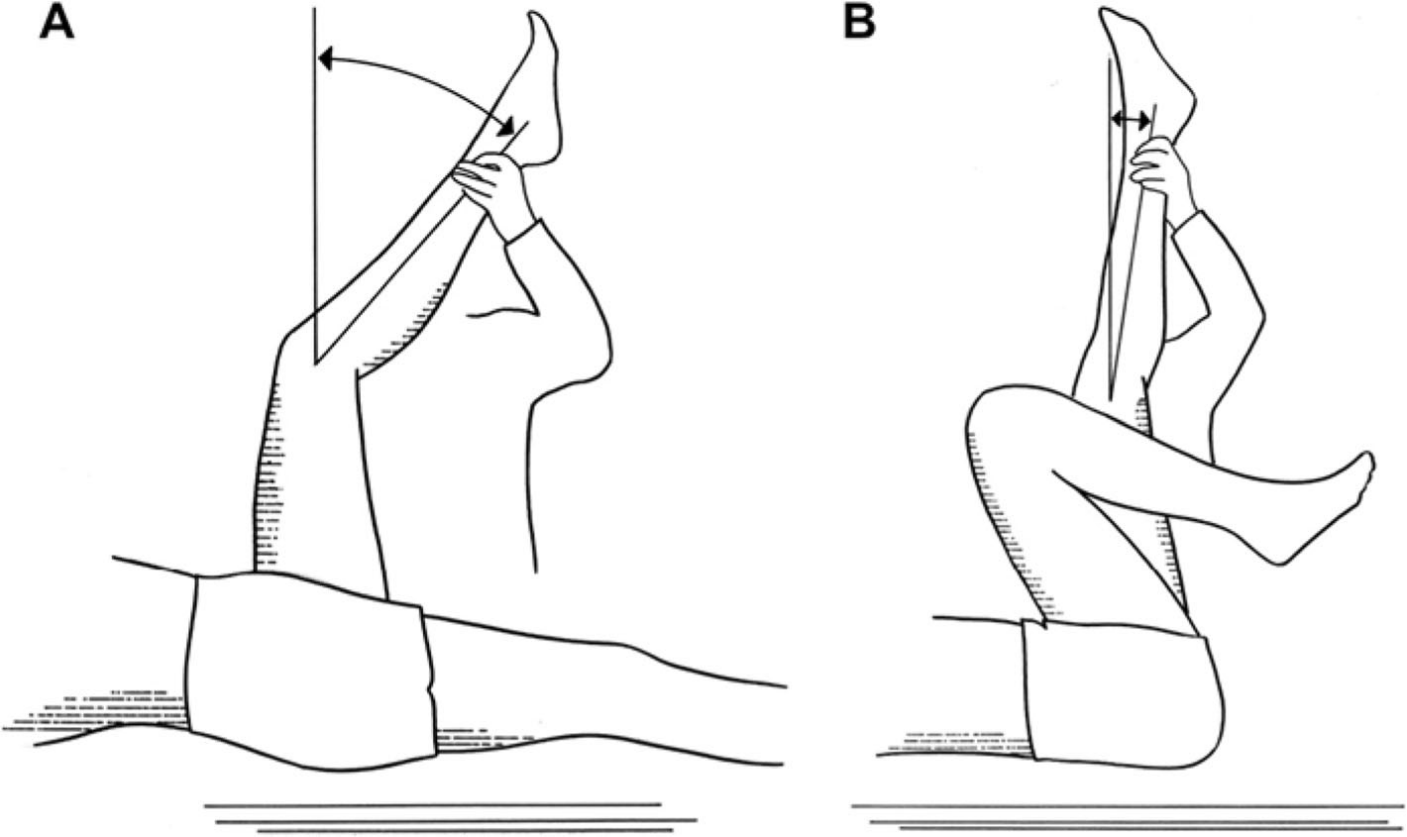
Tests to measure the popliteal angle. Unilateral test (**a**) and bilateral test (**b**). Reproduced with permission from ‘Orthopaedic Management in Cerebral Palsy’ by Eugene E. Bleck published by Mac Keith Press (www.mackeith.co.uk) in its Clinics in Developmental Medicine Series, 1987, 0–632–01523-3

The unilateral test was performed with the child in the supine position on the examination table. The examiner held the test leg in 90 degrees of hip flexion and the contralateral leg was fully extended and fixed. The examiner increased the knee extension until the leg was maximally passively extended. The movement was performed slowly (> 5 s for the entire range of motion) and with as much force as required to reach the passive end of motion. Reference marks for the goniometer ran along the femur to the greater trochanter and along the tibia to the lateral malleolus. The bilateral test was performed in a similar way as the unilateral test except that the child’s contralateral leg was flexed at the hip to a position in which the anterior superior iliac spine and the posterior superior iliac spine were vertical (Fig. 1).

For both tests, the goniometric measurement of the popliteal angle was defined as the angle, in degrees, of the lower leg relative to full extension (where 0 degrees was equal to full extension). The angle was recorded in 5-degree intervals as 0, 5, 10 or 15 degrees.

### Statistical analyses

The interrater reliability was evaluated using the intraclass correlation coefficient (ICC) and 95% confidence interval (CI) with two-way random and absolute agreement for single measures [15]. The ICC takes the degree of disagreement into account and should exceed 0.75 [16]. The ICC was estimated for the left and right sides separately and for the average value of the left and right side of each child. All analyses were performed by independent physiotherapists with no other knowledge of the participants using IBM SPSS Statistics (version 25).

## Results

In total, 70 participants (38 males and 32 females) with cerebral palsy were included. Their mean age was 10.8 years (range 5–22 years). The participants were classified according to the Gross Motor Function Classification System (GMFCS) level as follows: I (*n* = 17); II (*n* = 31); III (*n* = 12) and IV (*n* = 10).

The mean popliteal angle was 31 degrees measured in the unilateral test and 15 degrees in the bilateral test, in which the pelvis is tilted slightly more posteriorly. The popliteal angle measured in both tests increased with higher GMFCS level (Table 1).

**Table 1:** Mean value, standard deviation (SD) and range for the unilateral and bilateral popliteal angle tests

GMFCS level (n)	Type of test	Examiner	Mean	SD	Range
I (17)	Unilateral	1	27.35	10.1	10–50
		2	25.59	10.66	10–50
	Bilateral	1	13.53	10.5	0–35
		2	13.38	9.68	0–35
II (31)	Unilateral	1	33.31	10.69	15–65
		2	31.86	9.83	15–50
	Bilateral	1	16.21	9.9	0–35
		2	14.92	10.49	0–35
III (12)	Unilateral	1	33.96	7.65	20–50
		2	32.5	8.66	20–50
	Bilateral	1	17.71	8.36	0–35
		2	17.50	8.26	0–35
IV (10)	Unilateral	1	32.0	10.12	15–55
		2	32.75	11.99	20–60
	Bilateral	1	14.75	7.86	0–25
		2	17.75	15.96	0–60

Popliteal angles of the two examiners presented separately for each level of the Gross Motor Function Classification System (GMFCS)

The mean popliteal angle for both tests also increased with older age and there was a tendency for slightly higher values in males than females (Table 2).

**Table 2:** Popliteal angle with mean value and standard deviation (SD) for age, sex and GMFCS

	Unilateral test Mean (SD)	Bilateral test Mean (SD)
**GMFCS**		
I	26.47 (10.17)	13.46 (9.9)
II	32.58 (10)	15.56 (10.02)
III	33.23 (7.84)	17.6 (8.13)
IV	32.38 (10.82)	16.25 (11.32)
**Sex**		
Female	29.14 (9.04)	12.81 (8.03)
Male	32.89 (10.54)	17.76 (10.61)
**Age group**		
0–6	28 (9.23)	11.63 (8.46)
7–12	30.64 (9.34)	15.03 (8.71)
13–18	32.98 (11.12)	17.31 (11.62)
19–22	47.5 (10.61)	33.75 (10.61)

Average values for the unilateral and bilateral popliteal angle tests of both examiners together presented for the different sub groups

There was a high interrater reliability, with ICCs of ≥0.80 for both the unilateral and bilateral measurements of the popliteal angle (Table 3).

**Table 3:** Interrater reliability for the unilateral and bilateral popliteal angle tests

	Unilateral test		Bilateral test	
	ICC	95% CI	ICC	95% CI
**Right**				
Single measure	0.80	0.67–0.87	0.82	0.72–0.88
**Left**				
Single measure	0.86	0.78–0.91	0.83	0.74–0.89

Intraclass Correlation Coefficients (ICC) with 95% Confidence Intervals (CI) for the right and the left leg

## Discussion

This study showed that both the unilateral and bilateral tests to measure the popliteal angle have high interrater reliability for measuring hamstring length in children and youth with cerebral palsy. The participants in this study represented different ages and GMFCS levels. All participants had bilateral cerebral palsy to ensure that only the affected legs were included. Previous studies have shown that measuring range of motion can be more difficult in children with cerebral palsy than in those without cerebral palsy [17, 18]. A previous study of 15 children with cerebral palsy by Ten Berge et al. [19] showed good intrarater reliability but low interrater reliability for the unilateral popliteal test.

The unilateral popliteal angle reported was lower in this study (mean 31 degrees) than in other studies. Choi et al. [20] reported a mean unilateral popliteal angle of 37 degrees in children with GMFCS I or II. Bell et al. [21] reported a mean angle of 28 or 59 degrees, in children with CP depending on walking function and White et al. [22] reported a mean of 57.6 degrees for 8 to 12-year-old children with CP but did not specify whether the test was bilateral or unilateral. None of the previous studies specified how much pressure the examiners applied to extend the knee. The examiners in our study were instructed to extend the child’s leg with as much pressure as tolerated by the child and to reach the end range of movement. The range of 5–60 degrees reported by Choi et al. [20] was similar to the 0–60 degrees observed in our study. This wide range highlights the variability of measurement of the popliteal angle in children with cerebral palsy, which would affect the mean value in a smaller study population. Also capsular contracture may affect the range of motion. Reduced popliteal angle is one criterion for surgery to lengthen the hamstrings, but this should not be the only criterion [19, 23, 24].

The unilateral and bilateral tests can yield different results, as measured in degrees, according to the position of the pelvis. Therefore, when reporting results, it is important to indicate the type of measurement used and to consider the pelvic position [12]. A more posteriorly tilted pelvis, as in the bilateral test, will automatically yield a smaller popliteal angle. The bilateral test allows for relaxation of the hip flexors of the contralateral leg [25] and may be easier to use when measuring children with hip flexion contractures. A study by Manikowska et al. [23] showed a greater muscle activation of the contralateral leg during the unilateral compared with the bilateral test. The authors suggested that this muscle activation may affect the result and, therefore, should be considered.

The speed at which the knee extends during measurement is also important. Choi et al. [20] used the sum of a fast speed and slow speed measure of the popliteal angle when considering both spasticity and mechanical resistance, which can be important when evaluating treatment [26]. The aim of our study was to compare the interrater reliability between two frequently used clinical test methods to measure the muscle length of the hamstrings.

The popliteal angle was measured with a goniometer by two independent physiotherapists on the same day for all participants to minimize the effects of true changes in muscle length over time. The ICC was used to evaluate interrater reliability, and the values for both tests exceeded the minimum of 0.75 recommended by Streiner et al. [16] for clinical tests.

One limitation of this study is the potential influence on the examiners by the first test when performing the second test. However, the two examiners performed their measurements independently of each other and with the same conditions. The examiners had no personal interest in the results of the study that may have affected their measurements.

Another limitation of our study is that no children at GMFCS level V were included. It is possible that children at GMFCS level V are more difficult to measure depending on the severity and number of contractures. The tests were performed passively in the supine position, and it is unlikely that this affected the measurements in this study. However, the lack of children at GMFCS level V likely affected the mean value obtained in the tests to measure popliteal angle.

Some authors have questioned the measurement of hamstring length and suggest that the popliteal angle is a substitute measure and not a true measure of hamstring length [24, 27]. Other ways of measuring hamstring length, for example software techniques, have been used to evaluate the results of hamstring lengthening in children with cerebral palsy [28, 29]. A study by Park et al. showed that hamstring length measured with musculoskeletal modelling software correlated with the results of popliteal angle measurement [27].

## Conclusions

The popliteal angle is part of the regular examination of children with cerebral palsy. Given the variability in measuring methods and results, it is important to also include other examinations, such as gait analysis, when considering potential treatments [29].

This study showed high interrater reliability for both the unilateral and bilateral tests to measure popliteal angle. The ICCs were high and did not differ significantly between the two methods. Our finding suggests that both tests are reliable methods of measuring popliteal angle in children and youth with cerebral palsy.

## Data Availability

The data that support the findings of this study are available from the corresponding author, upon reasonable request.

## Abbreviations

CP: Cerebral palsy
CI: Confidence interval
GMFCS: Gross Motor Function Classification System
ICC: Intraclass correlation coefficient
